# Inferring changes in histone modification during cell differentiation by ancestral state estimation based on phylogenetic trees of cell types: Human hematopoiesis as a model case

**DOI:** 10.1016/j.gene.2019.100021

**Published:** 2019-05-31

**Authors:** Kanako O. Koyanagi

**Affiliations:** Faculty of Information Science and Technology, Hokkaido University, North 14, West 9, Kita-ku, Sapporo 060-0814, Japan

**Keywords:** B, B cell, BED, browser extensible data, ChIP-seq, chromatin immunoprecipitation sequencing, CRISPR, clustered regularly interspaced short palindromic repeat, DNA, deoxyribonucleic acid, Eo, eosinophil, Er, erythroblast, H3K4me1, monomethylation of histone H3 at lysine 4, H3K4me3, trimethylation of histone H3 at lysine 4, H3K9me3, trimethylations of histone H3 at lysine 9, H3K27ac, acetylation of histone H3 at lysine 27, H3K27me3, trimethylations of histone H3 at lysine 27, H3K36me3, trimethylation of histone H3 at lysine 36, kb, kilobase(s), KEGG, Kyoto encyclopedia of genes and genomes, L, lymphoid lineage, M, myeloid lineage, Me, megakaryocyte, Mo, monocyte, Ne, neutrophil, Nk, natural killer cell, T, T cell, TSS, transcription start sites, Ancestral state estimation, Cell lineage, Cell-type tree, Histone modification, Phyloepigenetics

## Abstract

Revealing the landscape of epigenetic changes in cells during differentiation is important for understanding the development of organisms. In this study, to infer such epigenetic changes during human hematopoiesis, ancestral state estimation based on a phylogenetic tree was applied to map the epigenomic changes in six kinds of histone modifications onto the hierarchical cell differentiation process of hematopoiesis using epigenomes of eight types of differentiated hematopoietic cells. The histone modification changes inferred during hematopoiesis showed that changes that occurred on the branches separating different cell types reflected the characteristics of hematopoiesis in terms of genomic position and gene function. These results suggested that ancestral state estimation based on phylogenetic analysis of histone modifications in differentiated hematopoietic cells could reconstruct an appropriate landscape of histone modification changes during hematopoiesis. Since integration of the inferred changes of different histone modifications could reveal genes with specific histone marks such as active histone marks and bivalent histone marks on each internal branch of cell-type trees, this approach could provide valuable information for understanding the cell differentiation steps of each cell lineage.

## Highlights

• Ancestral state estimation was used to infer epigenetic changes in cells during hematopoiesis.
• Six histone modification types and eight hematopoietic cell types were used.
• The landscape of histone modification changes could be reconstructed in terms of genomic position and gene function.

## 1 Introduction

Cell differentiation is regulated by transcription factors and epigenetic modifications, which influence gene expression patterns. To understand the process of cell differentiation, it is important to know the changes occurring in gene expression and epigenetic patterns of each cell lineage. However, comprehensively analyzing such changes in each cell lineage by experiments is laborious, and sometimes impossible, especially *in vivo*. Epigenetic changes that occur throughout cell differentiation might represent key features of the differentiation processes. Indeed, nucleosome patterns are suggested to represent cell types of hematopoietic cells better than gene expression patterns (Corces et al., 2016) likely because of the variable features of gene expression. Recently, it has been shown that phylogenetic analyses of DNA methylation patterns in differentiated cells can be used to infer hierarchical cell differentiation processes and the DNA methylation status of differentiating progenitor cells (Capra and Kostka, 2014; Koyanagi, 2015). Similar to phylogenetic trees that are used to analyze evolutionary relationships, cell differentiation pathways also form a tree, known as a cell-type tree, which represents the cell differentiation hierarchy (Kin, 2015). Thus, the same approaches employed to construct evolutionary trees can be used to infer cell differentiation processes. Especially, ancestral state estimation based on phylogenetic analysis has an advantage over other methods used to compare epigenomes of different cell types, such as the clustering method, because it allows inference of not only tree topology, corresponding to the cell differentiation hierarchy, but also ancestral states, corresponding to the epigenetic states of the differentiating progenitor cells. By comparing the epigenomes of adult differentiated cells, we could infer the epigenome landscape of each cell lineage.

Like DNA methylation, histone modifications present another important type of epigenetic alteration. While DNA methylation mainly represses gene expression (Luo et al., 2018), histone modification is involved in both the activation and repression of gene expression (Zhou et al., 2011; Lawrence et al., 2016). Several types of histone modifications, including methylation, acetylation, phosphorylation, and ubiquitination at different sites of histone molecules, regulate their interaction with DNA and influence gene transcription. For example, monomethylation of histone H3 at lysine 4 (H3K4me1) and acetylation of histone H3 at lysine 27 (H3K27ac) in enhancer regions are associated with transcriptional activation, and trimethylation of histone H3 at lysine 4 (H3K4me3) and H3K27ac in promoter regions are also involved in transcriptional activation (Zhou et al., 2011; Lawrence et al., 2016). Further, trimethylation of histone H3 at lysine 36 (H3K36me3) in gene body regions is associated with transcription elongation, and trimethylations of histone H3 at lysine 27 (H3K27me3) and at lysine 9 (H3K9me3) are associated with facultative and constitutive transcriptional repression, respectively (Zhou et al., 2011; Lawrence et al., 2016). Therefore, genome-wide changes in histone modification provide clues about cell differentiation processes, which are regulated by gene expression (Meissner, 2010; Dambacher et al., 2013; Boland et al., 2014; Avgustinova and Benitah, 2016).

Although how histone modifications are inherited through cell division is not as fully understood as is DNA methylation, histone modifications are stable during cell division (Almouzni and Cedar, 2016). Thus, phylogenetic analysis of histone modification could be useful for inferring the epigenome landscape in differentiating cells. Based on the histone modification data from ENCODE project (ENCODE Project Consortium, 2012), which sampled various cell types from the human body, Nair et al. showed that phylogenetic analysis can build biologically meaningful cell-type trees, in which cell types of the same origin are clustered in the phylogenetic tree (Nair et al., 2014; Nair, 2015). In this study, using ancestral state estimation based on phylogenetic trees of differentiated hematopoietic cells, six kinds of histone modifications of human hematopoietic cells were mapped onto the hierarchical cell differentiation process, in which histone modification plays an important role (Avgustinova and Benitah, 2016; Carrillo-de-Santa-Pau et al., 2017; Raghuwanshi et al., 2018). The aim of this study was to examine whether ancestral state estimation of histone modifications could be used to reconstruct the changing history of histone modification during hematopoiesis.

## 2 Materials and methods

### 2.1 Histone modification data

Genome-wide histone modification data for human hematopoietic cells were obtained from the latest eighth data release of the BLUEPRINT project (ftp://ftp.ebi.ac.uk/pub/databases/blueprint/). This dataset contains ChIP-seq analysis data for various histone modifications in hematopoietic cells, and the genomic regions with each histone modification are available in Browser Extensible Data (BED) format. For this study, all the available data of differentiated cells in blood having six histone modifications (H3K4me1, H3K4me3, H3K27ac, H3K36me3, H3K27me3, and H3K9me3) were used, which were derived from eight types of differentiated cells (erythroblasts, megakaryocytes, eosinophils, neutrophils, monocytes, natural killer cells, T cells, and B cells) in blood. The data contain cells of the same type obtained from different tissues (venous blood, cord blood, tonsil, and bone marrow) and from different individuals (Supplementary Table 1). Samples whose ChIP-seq data are missing for at least one chromosome (except Y) were excluded from the analyses.

### 2.2 Ancestral state estimation based on phylogenetic analyses

Based on the BED files, genomic sites (nucleotides) with ChIP-seq signals were regarded as histone modification “ON”, and all other sites were regarded as histone modification “OFF”. Based on these binary ON/OFF sites corresponding to individual nucleotides, phylogenetic analyses were performed using the maximum parsimony method with PAUP 4.0 (Swofford, 2003). The character type was treated as undirected, with the cost of modification of “ON” being equal to that of “OFF”. Monophyly of the same cell types and the traditional hierarchical differentiation model, which is (((erythroblasts, megakaryocytes), ((eosinophils, neutrophils), monocytes)), (natural killer cells, T cells, B cells)), were assumed, although the traditional hierarchical model of hematopoiesis has become a matter of debate owing to recent technical advances (Laurenti and Göttgens, 2018). Other unfixed phylogenetic relationships were inferred by heuristic search with tree bisection-reconnection algorithm. The ancestral state for each node was inferred with accelerated transformation (ACCTRAN) and delayed transformation (DELTRAN) algorithms (Swofford and Maddison, 1987). Because results obtained from both algorithms were similar, those of ACCTRAN are shown in the main text.

### 2.3 Annotation analyses

To characterize histone modification sites, the ChIPseeker package (Yu et al., 2015) of R/Bioconductor was used with TxDb.Hsapiens.UCSC.hg38.knownGene as the human genome annotation database. Using the annotate Peak function of ChIPseeker, the genomic location of histone modification relative to transcription start sites (TSS) was assigned. To test genomic distribution differences between histone modifications on inter-type branches and those on intra-type branches, Kolmogorov–Smirnov test was performed using ks.test function in R (3.4.1). For histone modifications associated with genes, functional enrichment analysis was performed using the compare cluster function of cluster Profiler package (Yu et al., 2012) of R/Bioconductor with KEGG pathway database (Kanehisa et al., 2004). *P* values <0.05 after correction with the Benjamini-Hochberg procedure were considered to represent significant enrichment. The intersection of inferred histone modification sites with known genes in the human genome was analyzed using BEDTools (Quinlan and Hall, 2010) with genome annotation of GENCODE (Harrow et al., 2012) Release 22 downloaded from ftp://ftp.ebi.ac.uk/pub/databases/blueprint/reference/.

## 3 Results and discussion

### 3.1 Inferred histone modification changes mapped onto a cell-type tree

**Fig. 1:**
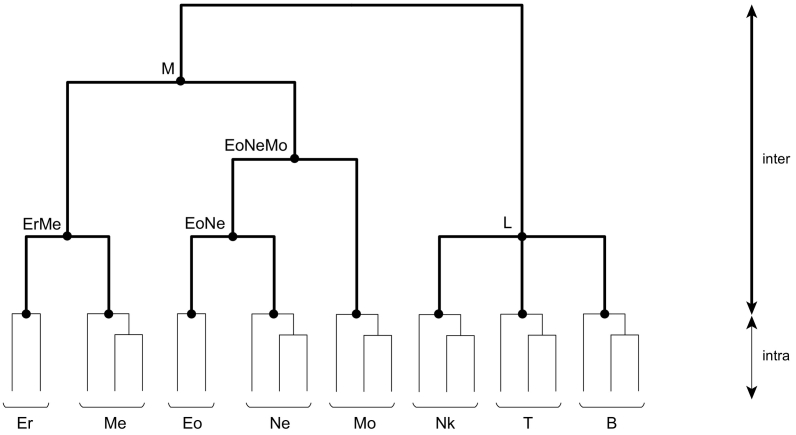
Differentiation model of hematopoiesis of eight cell types used in this study Thick and thin lines indicate inter-type and intra-type branches, respectively (see text). M, myeloid lineage; L, lymphoid lineage; Er, erythroblast; Me, megakaryocyte; Eo, eosinophil; Ne, neutrophil; Mo, monocyte; Nk, natural killer cell; T, T cell; B, B cell.

Human hematopoiesis, one of the best-studied cell differentiation processes, was used as the model case in this study. Hematopoietic stem cells give rise to myeloid and lymphoid lineages, producing various types of differentiated cells (Laurenti and Göttgens, 2018) (Fig. 1). For eight types of differentiated hematopoietic cells (erythroblasts, megakaryocytes, eosinophils, neutrophils, and monocytes of the myeloid lineage, and natural killer cells, T cells, B cells of the lymphoid lineage), epigenome information regarding six histone modifications (H3K4me1, H3K4me3, H3K27ac, H3K36me3, H3K27me3, and H3K9me3) was obtained from ChIP-seq data of the BLUEPRINT project (Fernández et al., 2016). The samples of each cell type were obtained from different tissues, individuals, and subtypes making a total of 114, 122, 118, 83, 99, and 104 samples available for H3K4me1, H3K4me3, H3K27ac, H3K36me3, H3K27me3, and H3K9me3, respectively (Supplementary Table 1). Genomic sites (nucleotides) with ChIP-seq signals were regarded as histone modification “ON” sites, and genomic sites without ChIP-seq signals were regarded as histone modification “OFF” sites. The ancestral states (ON/OFF histone modification) at each site for each node for each histone modification were inferred based on a fixed tree topology, which corresponds to a well-known traditional hierarchical differentiation process (see Materials and methods for details; the tree topologies used for the inference for the six histone modifications are shown in Supplementary Fig. 1). In this study, branches were classified as either inter-type or intra-type branches (Fig. 1). Inter-type branches separate the eight hematopoietic cell types, while intra-type branches separate the same cell types obtained from different tissues and/or individuals as well as different subtypes of the same cell type (Supplementary Table 1). For the inference, the maximum parsimony method was used similar to that in previous studies where cell-type trees were inferred based on histone modification (Nair et al., 2014) and gene expression (Joshi and Göttgens, 2011; Kin et al., 2015). As a result, 878,349,583, 154,715,602, 388,431,451, 635,179,539, 932,000,779, and 1,048,771,872 variable sites (nucleotides) were available for H3K4me1, H3K4me3, H3K27ac, H3K36me3, H3K27me3, and H3K9me3, respectively, and the history of histone modification changes at each site for each branch was mapped onto the trees (the result of inter-type branches is shown in Table 1). The result showed that overall histone modification changing patterns differed between the histone modification types and cell lineages (branches).

### 3.2 Genomic locations of histone modification changes occurring on inter- and intra-type branches

**Fig. 2:**
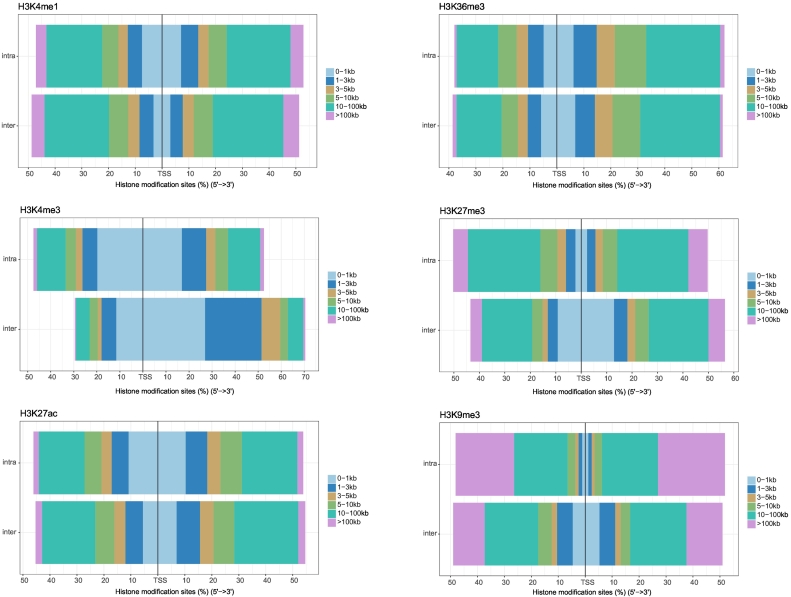
Distribution of histone modification changes relative to transcription start sites The horizontal axis represents the proportion of sites where histone modification changes occurred relative to transcription start sites (TSS) of the nearest gene. The left half corresponds to the sites upstream of TSS, while the right half corresponds to the sites downstream of TSS. The color indicates the genomic position of the histone modification sites relative to TSS.

To examine whether the histone modification changes during hematopoiesis could be inferred appropriately, the genomic locations of histone modification changes were compared between those occurring only on inter-type branches and those occurring only on intra-type branches. For each histone modification site, the distance from the TSS of the nearest gene was calculated and classified into 12 categories (0–1 kb, 1–3 kb, 3–5 kb, 5–10 kb, 10–100 kb, and > 100 kb for both upstream and downstream from TSS). The percentage (sum of sites divided by total sites) for each category is shown in Fig. 2. The distribution showed different genomic locations relative to TSS between inter- and intra-type branches. The differences were significant (Kolmogorov–Smirnov test, *P* values <2.2e-16) for all the histone modifications except for H3K36me3. Enrichment of H3K4me3 and H3K27me3 near TSS was found on inter-type branches in comparison with that on intra-type branches. Because H3K4me3 and H3K27me3 are known to be overrepresented in the promoter regions of active and repressed genes, respectively (Zhou et al., 2011), these results showed that these histone modification changes inferred to occur on inter-type branches reflected the roles of the histone modification as a regulator of cell-type differentiation. Especially, H3K4me3 was enriched in downstream of TSS (Fig. 2), consistent with a previous report (Barski et al., 2007). H3K4me1 showed slight enrichment in distal regions to TSS on inter-type branches compared with intra-type branches, possibly because it is known to be enriched in active enhancer regions, while H3K36me3 did not show any tendency related to TSS, likely because it is known to bind active gene body regions (Zhou et al., 2011). Overall, these results suggested that the ancestral state estimation could reconstruct histone modification changes on inter-type branches, whose genomic distribution showed characteristic of each histone modification. The histone modifications inferred on intra-type branches might contain modifications related to environmental and individual variation (Chen et al., 2016; Cheung et al., 2018) and modifications related to function other than transcriptional regulation, such as DNA repair (Wei et al., 2018), which might weaken the characteristic genomic distribution of each kind of histone modification.

**Table 1:** The number of histone modification changes (nucleotids) for each inter-type branch.

Modification	Change	Inter-type branch^a^											
		M-L	M-ErMe	M-EoNeMo	ErMe-Er	ErMe-Me	EoNeMo-EoNe	EoNe-Eo	EoNe-Ne	EoNeMo-Mo	L-Nk	L-T	L-B
H3K4me1	ON	9,429,466	39,703,057	22,017,758	35,913,942	34,886,942	24,421,323	25,468,079	26,236,718	19,461,601	33,753,469	27,581,198	33,533,882
	OFF	18,781,513	9,462,402	8,422,473	2,591,383	7,911,598	6,476,176	11,206,242	5,179,445	10,922,953	22,154,142	22,625,207	5,541,398
H3K4me3	ON	1,684,125	991,061	1,313,708	179,792	661,888	795,410	182,598	2,285,809	2,941,744	1,409,903	613,327	5,859,569
	OFF	415,222	2,565,055	563,361	570,719	12,546	1,790,294	2,871,781	105,427	115,196	2,734,433	2,227,015	93,935
H3K27ac	ON	1,139,495	3,438,861	2,601,470	3,244,347	2,338,903	1,414,498	18,385	5,776,718	2,678,442	15,562,073	9,348,850	962,543
	OFF	587,997	914,490	1,041,058	340,141	160,301	748,342	1,998,988	22,581	395,810	467,148	340,135	636,148
H3K36me3	ON	9,220,171	20,703,797	11,042,540	13,320,114	14,424,860	10,380,855	6,095,266	16,615,215	10,233,806	10,266,062	15,715,811	20,588,130
	OFF	11,278,817	13,598,172	10,329,194	5,514,091	5,530,871	10,859,870	13,945,648	3,327,696	6,078,638	24,007,727	13,181,707	4,574,464
H3K27me3	ON	640,647	3,548,907	3,848,679	4,699,466	0	12,056,241	7,385,629	7,662,155	2,485,809	5,584,214	911,630	1,721,789
	OFF	6,597,173	1,447,281	554,443	0	2,288,655	367,596	1,521,212	493,840	3,222,529	886,857	7,829,706	8,627,963
H3K9me3	ON	34,296	12,086,241	1,654,719	19,002,388	88	6,152,032	14,785,266	1,622,564	1,109,078	441,776	872,060	506,071
	OFF	13,774,405	126,221	203,243	346	686,622	1,030,277	385,000	2,544,737	2,620,331	5,127,667	3,316,966	1,646,179

a Inter-type branches are shown with nodes. For example, M-L indicates the inter-type branch which connects myeloid lineage (the ancestral node of Er, Me, Eo, Ne, and Mo) and lymphoid lineage (the ancestral node of Nk, T, and B). Abbreviations are the same as Fig. 1.

### 3.3 Functions of genes with histone modification changes occurring on inter- and intra-type branches

**Fig. 3:**
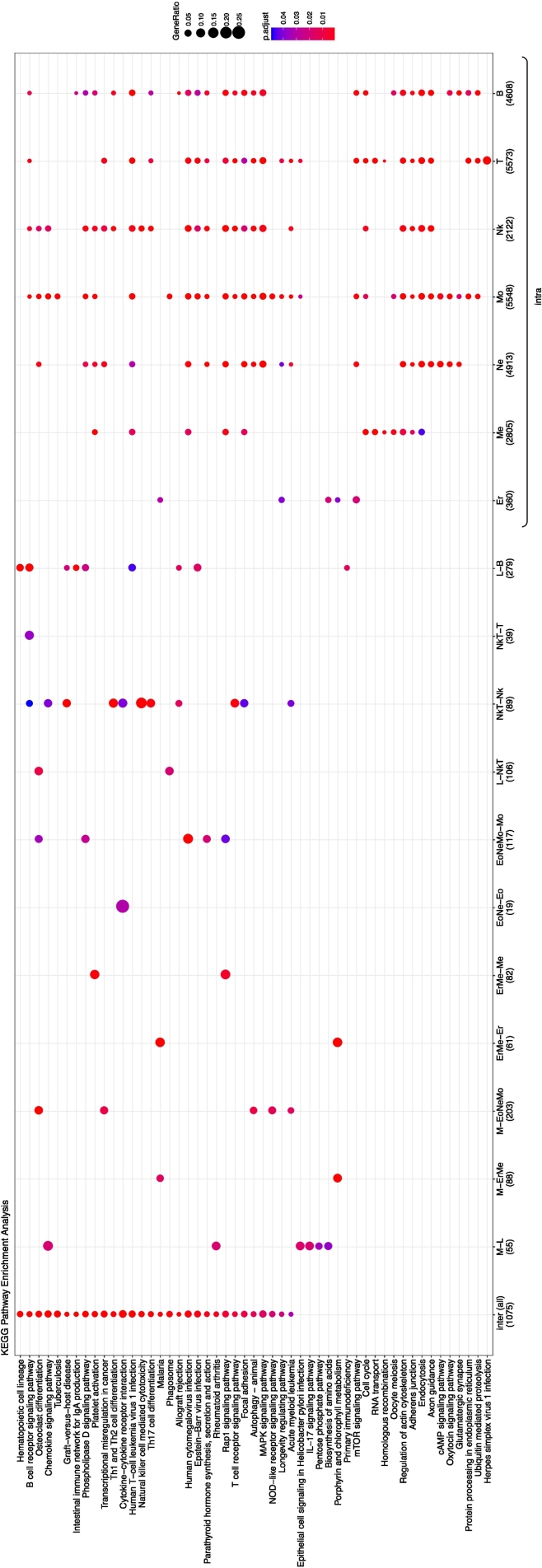
Functional annotation of genes with H3K4me3 changes (ON) for each branch Enriched functional categories of Kyoto Encyclopedia of Genes and Genomes (KEGG) are shown for inter-type branches and intra-type branches of each cell type. “Inter (all)” indicates the changes occurring only on inter-type branches. Inter-type branches were also analyzed separately and shown. Gene ratio is represented as a circle, and its color corresponds to adjusted *P* value that was corrected based on Benjamini-Hochberg method for multiple testing. Numbers in parentheses are the number of genes. Enriched functional category was not detected in eosinophils and is thus not shown. Abbreviations are the same as Fig. 1 and Table 1.

To further characterize histone modification changes occurring only on inter- or intra-type branches, the functional annotations of genes associated with histone modifications were analyzed. The results showed differences in the histone modification changes observed on inter- and intra-type branches (Fig. 3 for H3K4me3 and Supplementary Fig. 2 for other modifications). For example, H3K4me3 changes were enriched in genes related to “hematopoietic cell lineage” only on inter-type branches (Fig. 3). Furthermore, when inter-type branches were analyzed separately, lineage-specific features emerged. For example, H3K4me3 changes only occurring on the inter-type branch connecting to megakaryocytes (ErMe-Me in Fig. 3) were enriched in “platelet activation”, reflecting the differentiation process of megakaryocytes. Similarly, H3K4me3 changes only occurring on the inter-type branch connecting to NK cells (NkT-Nk in Fig. 3) were enriched in “natural killer cell mediated cytotoxicity” while those only occurring on the inter-type branches connecting to erythroblasts (M-ErMe and ErMe-Er in Fig. 3) were enriched in “porphyrin and chlorophyll metabolism”, reflecting hemoglobin synthesis. In contrast, H3K4me3 changes only occurring on intra-type branches of each cell type showed less cell-lineage specific function, where functional enrichment was not exclusively observed in an expected cell type, which might be consistent with the result of subsection 3.2 showing that intra-type branches included histone modifications other than those involving transcriptional regulation. As a whole, the results suggested that ancestral state estimation could reconstruct hematopoiesis-related histone modification changes that reflected the specific function of cell lineage at least for inter-type branches and partly for intra-type branches.

### 3.4 Inferred histone marks during hematopoiesis

Integration of different kinds of inferred histone modifications could be useful for revealing the genes involved in cell differentiation processes for each branch. For example, H3K4me3 and H3K27ac modifications in promoter regions are known as “active marks” related to the activation of gene expression, while H3K4me3 and H3K27me3 modifications are known as “bivalent” having both activating and repressive chromatin found in progenitor cells (Zhou et al., 2011). Table 2 shows the example of *GATA-1* gene, which encodes a transcription factor involved in erythroid cell and megakaryocyte development (Wolff and Humeniuk, 2013). H3K4me3 and H3K27ac modifications, found in erythroblast and megakaryocyte lineages, were experimentally shown in mice (Attema et al., 2007; Lara-Astiaso et al., 2014), indicating that ancestral estimation could correctly infer active marks of differentiating progenitor cells (internal nodes) for this gene. Since genome-wide histone modification information for human differentiating progenitor cells such as common lymphoid progenitors, common myeloid progenitors, granulocyte-macrophage progenitors, and megakaryocyte-erythrocyte progenitors is not publicly available currently, inferred combinatorial pattern of histone marks for each gene of internal nodes, corresponding to those progenitor cells, could be valuable resources (available as Supplementary Table 2) for future research to understand the gene regulation in each cell lineage during hematopoiesis.

As shown in Supplementary Table 1 and Supplementary Fig. 1, each cell type contains a heterogeneous population of cells from different tissues, individuals, and subtypes. It should be noted that the monophyly of the same cell types and the traditional hierarchical differentiation orders were assumed (fixed) in this study to obtain an average profile of histone modification changes occurring through hematopoiesis. Although the obtained results showed the reasonable inference of histone modification changes, this approach has some limitations. For example, it has been shown recently that granulocyte–monocyte progenitors are heterogeneous and they can be derived from both myeloid and lymphoid lineages (Laurenti and Göttgens, 2018). In such cases, this study can infer changing events assigned to an incorrect branch. Therefore, further analyses are needed to obtain a detailed and precise epigenetic landscape of hematopoiesis in future studies.

Cell lineage, corresponding to cell division history, has been revolutionized by newly emerged technologies (Shapiro et al., 2013; Woodworth et al., 2017). For example, single-cell genomics with CRISPR gene editing techniques and subsequent phylogenetic analysis revealed cell lineage trees of zebrafish (McKenna et al., 2016) and mice (Kalhor et al., 2018). Furthermore, single-cell transcriptomics can infer pseudotime ordering of cells during development–differentiation trajectories (Fletcher et al., 2018; Kester and van Oudenaarden, 2018). These new technologies also led to an updated view of differentiation landscapes of hematopoietic cells (Höfer et al., 2016; Giladi and Amit, 2018; Laurenti and Göttgens, 2018). Because cell lineage can differ from cell differentiation trajectories (Marioni and Arendt, 2017), integrative analyses of these cell lineage trees, cell differentiation trajectories and epigenetic landscape, inferred here for human hematopoiesis as a model case, would enable a deeper understanding of the relationship between differentiation of diverse cell types and the biological processes underlying the development of an organism.

**Table 2:** The number of histone modification sites (nucleotides) of *GATA-1* gene inferred for each internal node.

Region	Modification^a^	Internal node^b^												
		M	ErMe	Er	Me	EoNeMo	EoNe	Eo	Ne	Mo	L	Nk	T	B
5′ upstream	H3K4me1	0	2644	2714	2644	0	0	2358	0	0	0	0	0	0
	H3K4me3	0	394	394	394	0	0	0	0	0	0	0	0	0
	H3K27ac	0	501	643	501	0	0	0	0	0	0	0	0	0
Gene	H3K4me3	0	2664	2664	2664	0	0	1389	0	0	0	0	0	0
	H3K27ac	0	1723	1798	2094	0	0	0	0	0	0	0	0	0
	H3K36me3	0	3587	3888	3587	0	0	2981	0	0	0	0	0	0

a Histone modification in which at least one node has a modification (> 0 sites) is shown.

b Abbreviations are the same as Fig. 1.

## 4 Conclusions

This study suggested that ancestral state estimation based on phylogenetic analysis of histone modifications could reconstruct the landscape of histone modification during hematopoiesis. The inferred histone modification changes during cell differentiation processes reflected the characteristics of hematopoiesis of each cell lineage in terms of genomic position and gene functions. This approach can be extended to other cell types to understand multiple cell differentiation processes other than hematopoiesis by inferring transitions of epigenomes.

The following are the supplementary data related to this article.

Supplementary Table 1 Samples used. Supplementary Table 1

Supplementary Table 2 The number of inferred histone modification sites (nucleotides) of each gene for each internal node. Supplementary Table 2

Supplementary Fig. 1 Phylogenetic trees used for the inference. Supplementary Fig. 1

Supplementary Fig. 2 Functional annotation of genes with histone modification changes (ON) for each branch. Supplementary Fig. 2
