# Supplementary figures and images for: Inferring changes in histone modification during cell differentiation by ancestral state estimation based on phylogenetic trees of cell types: Human hematopoiesis as a model case
Source: Gene X. 2019 May 31;3:100021. doi: 10.1016/j.gene.2019.100021 (PMC7286071; doi:10.1016/j.gene.2019.100021)

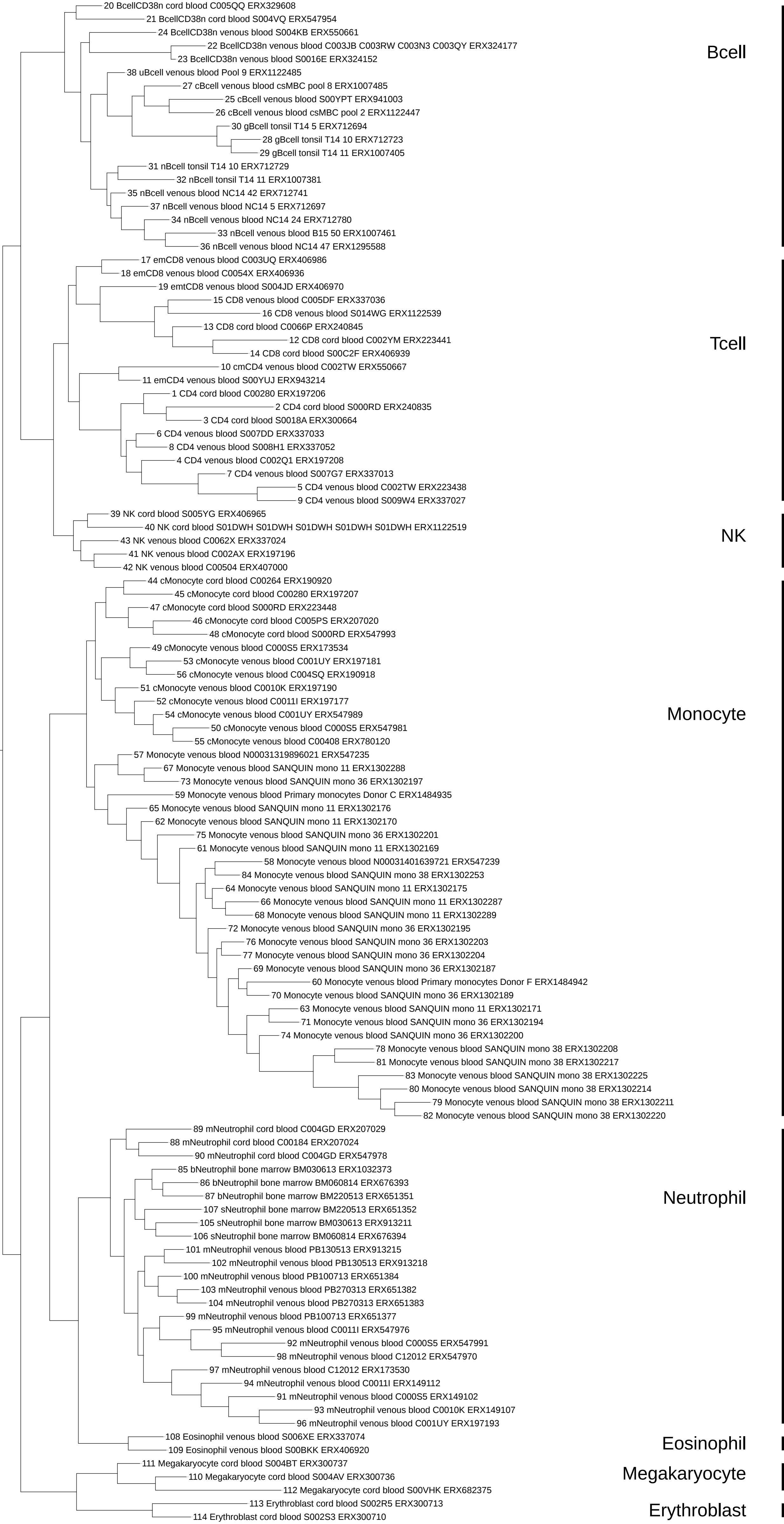

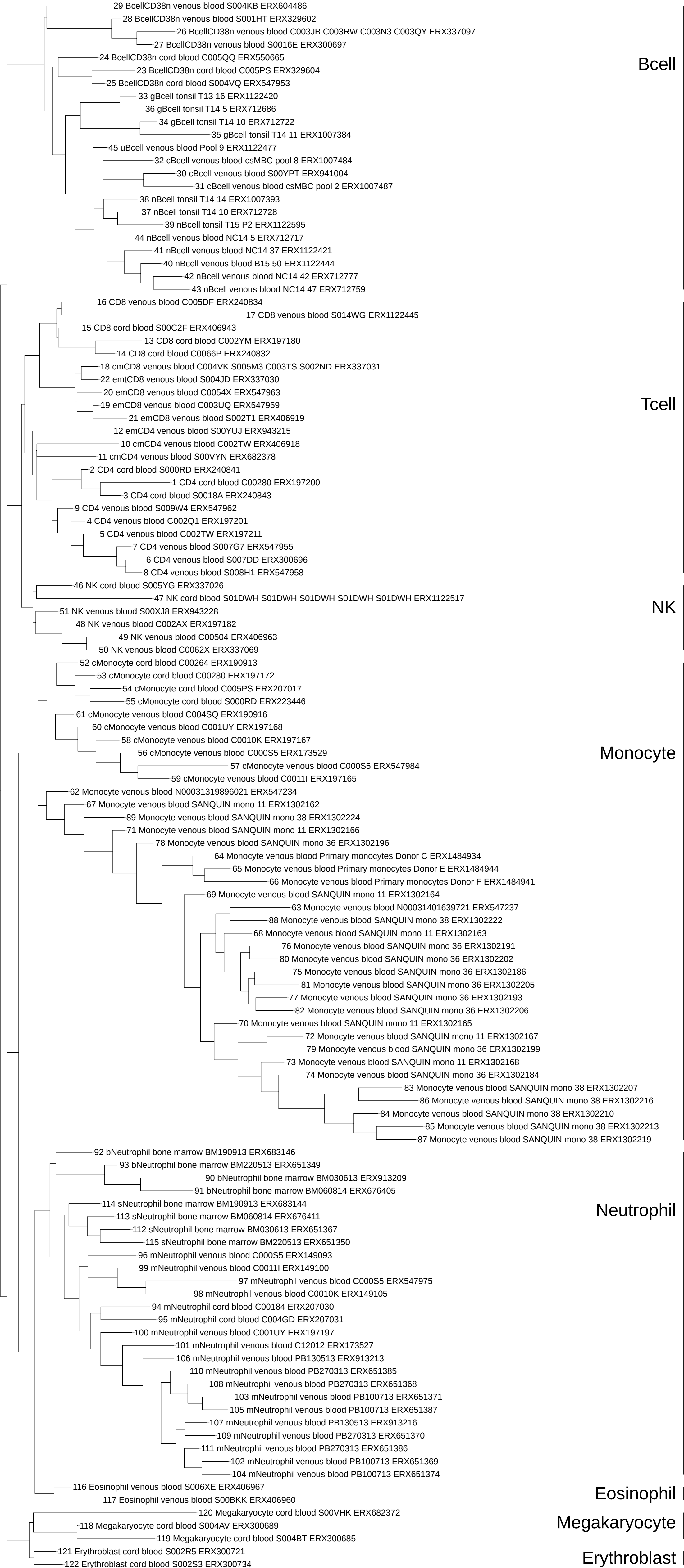

H3K27ac

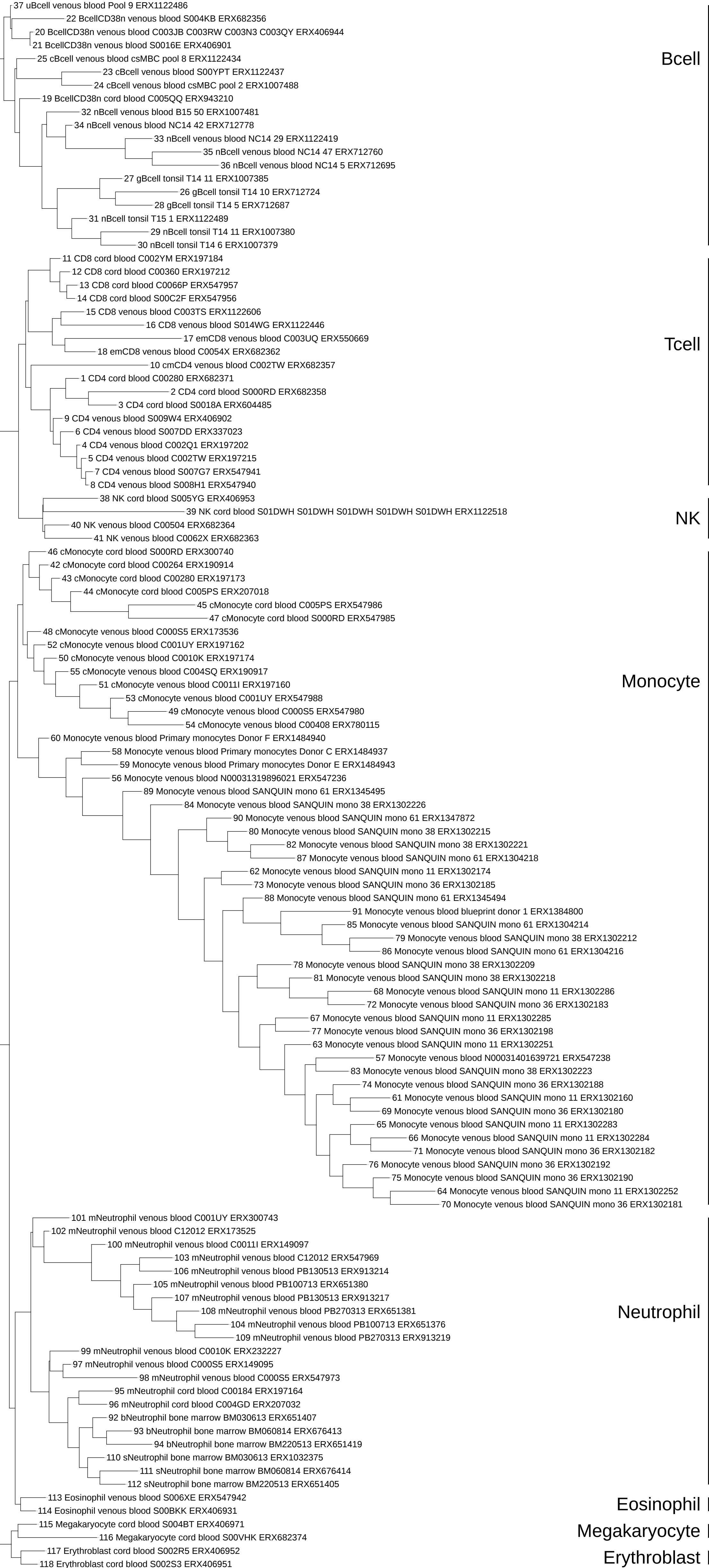

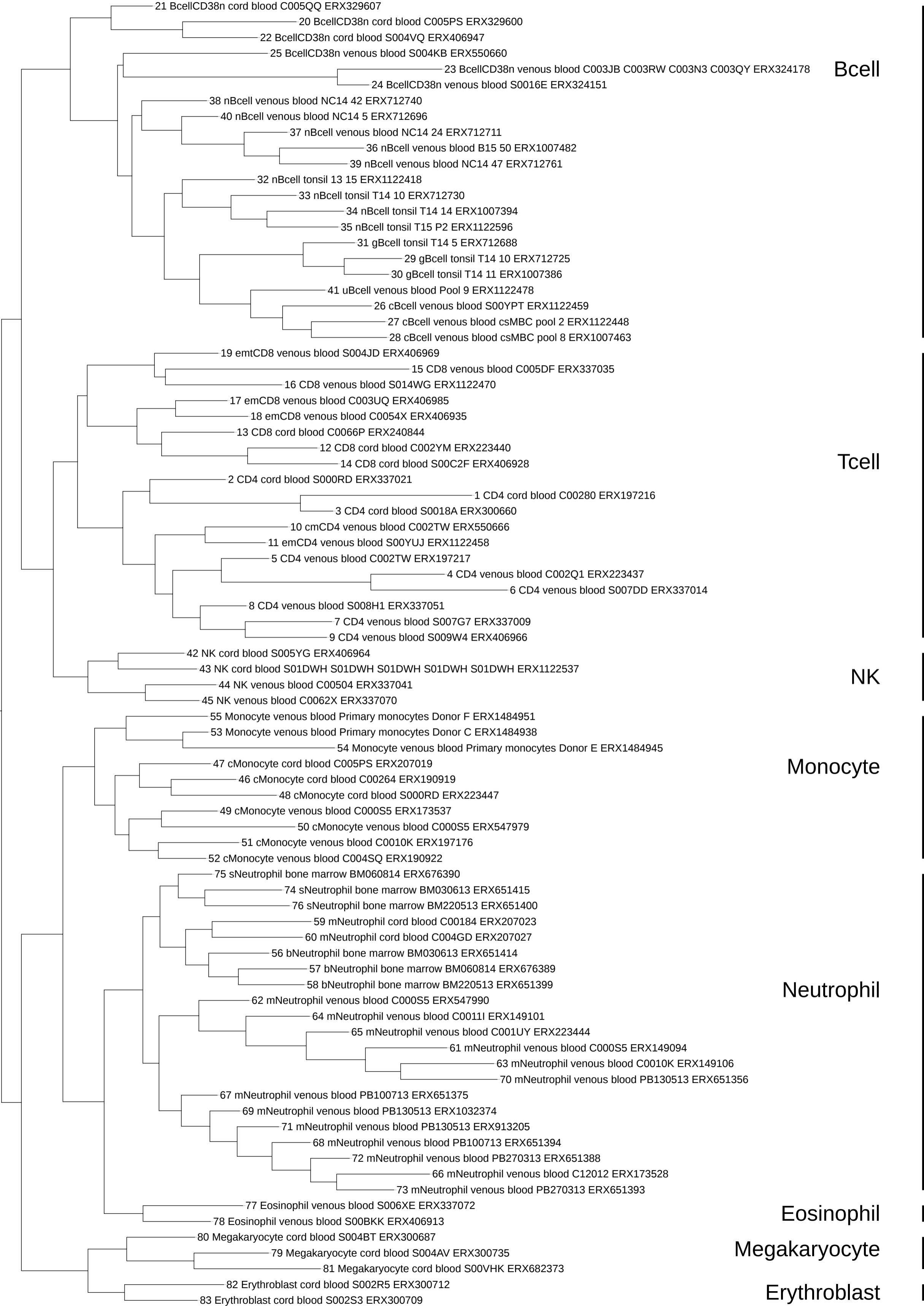

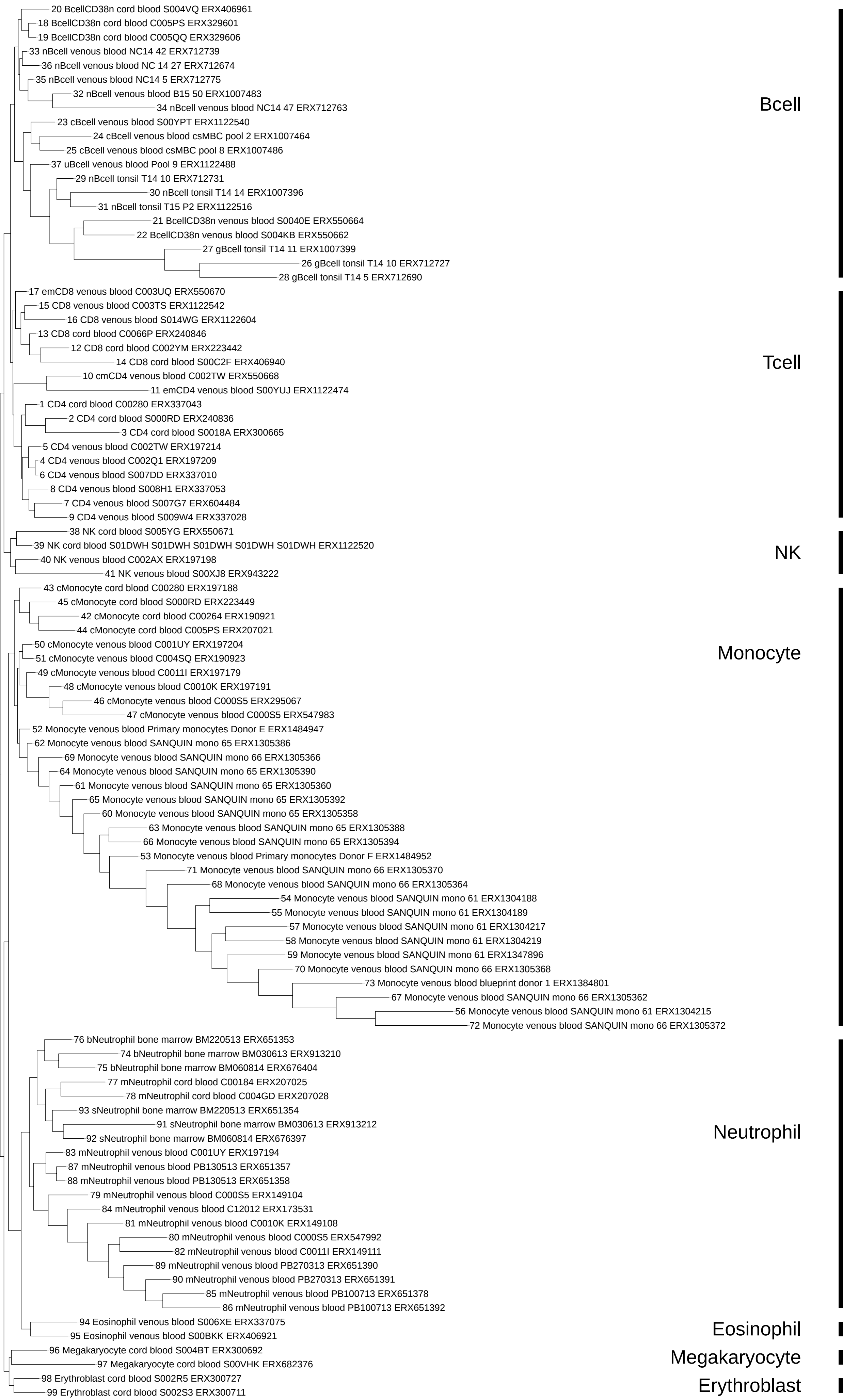

# H3K9me3

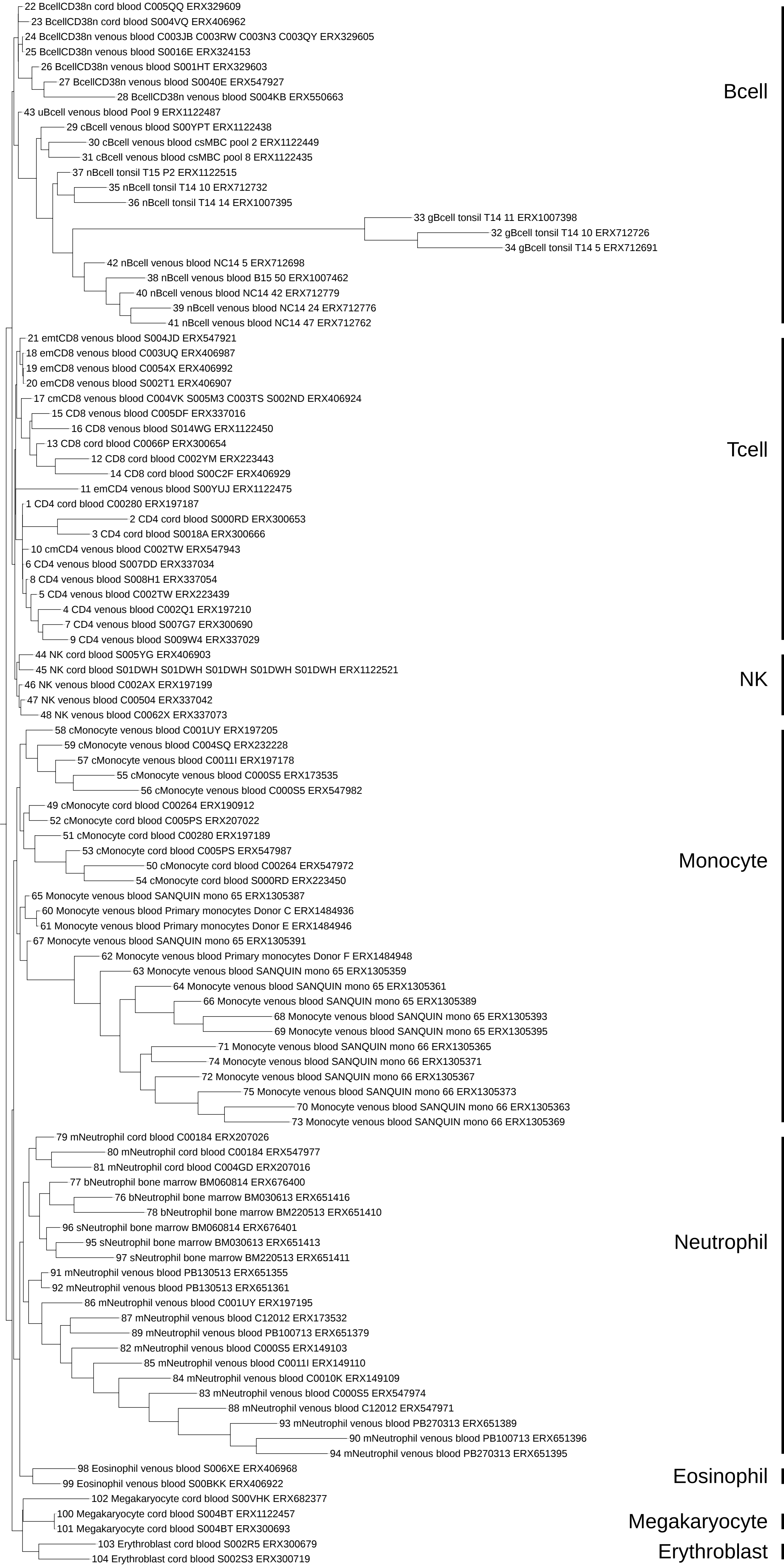

Supplement: Supplementary Fig. 1 — Phylogenetic trees used for the inference. [file mmc3.pdf]

H3K4me1

KEGG Pathway Enrichment Analysis

Supplementary Figure 2

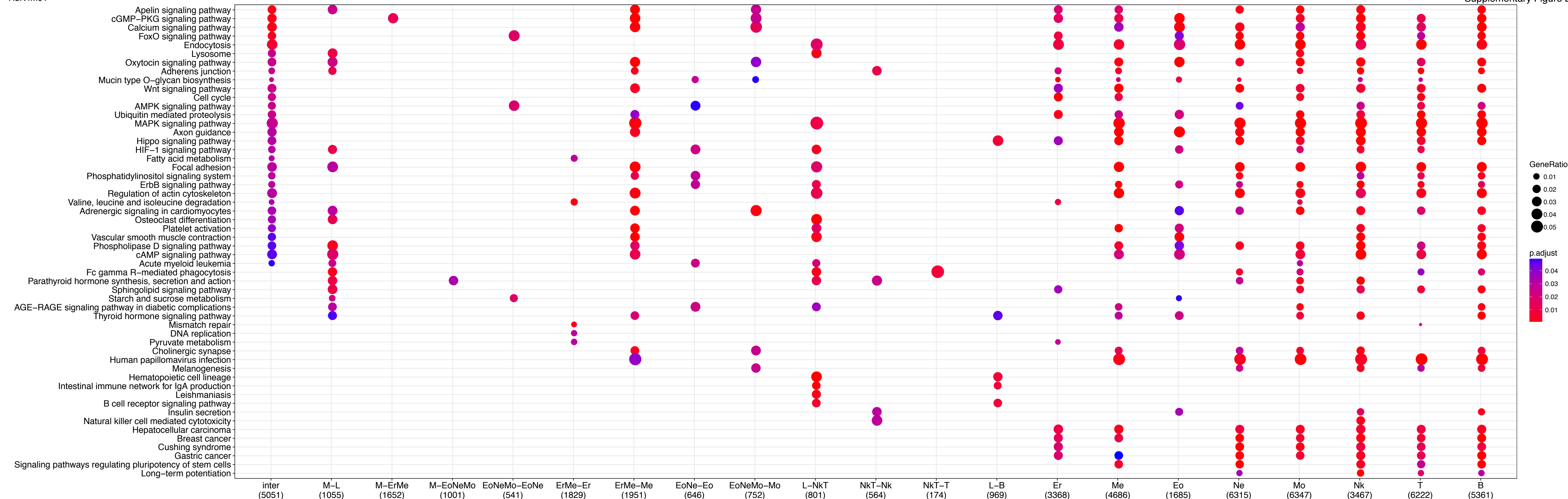

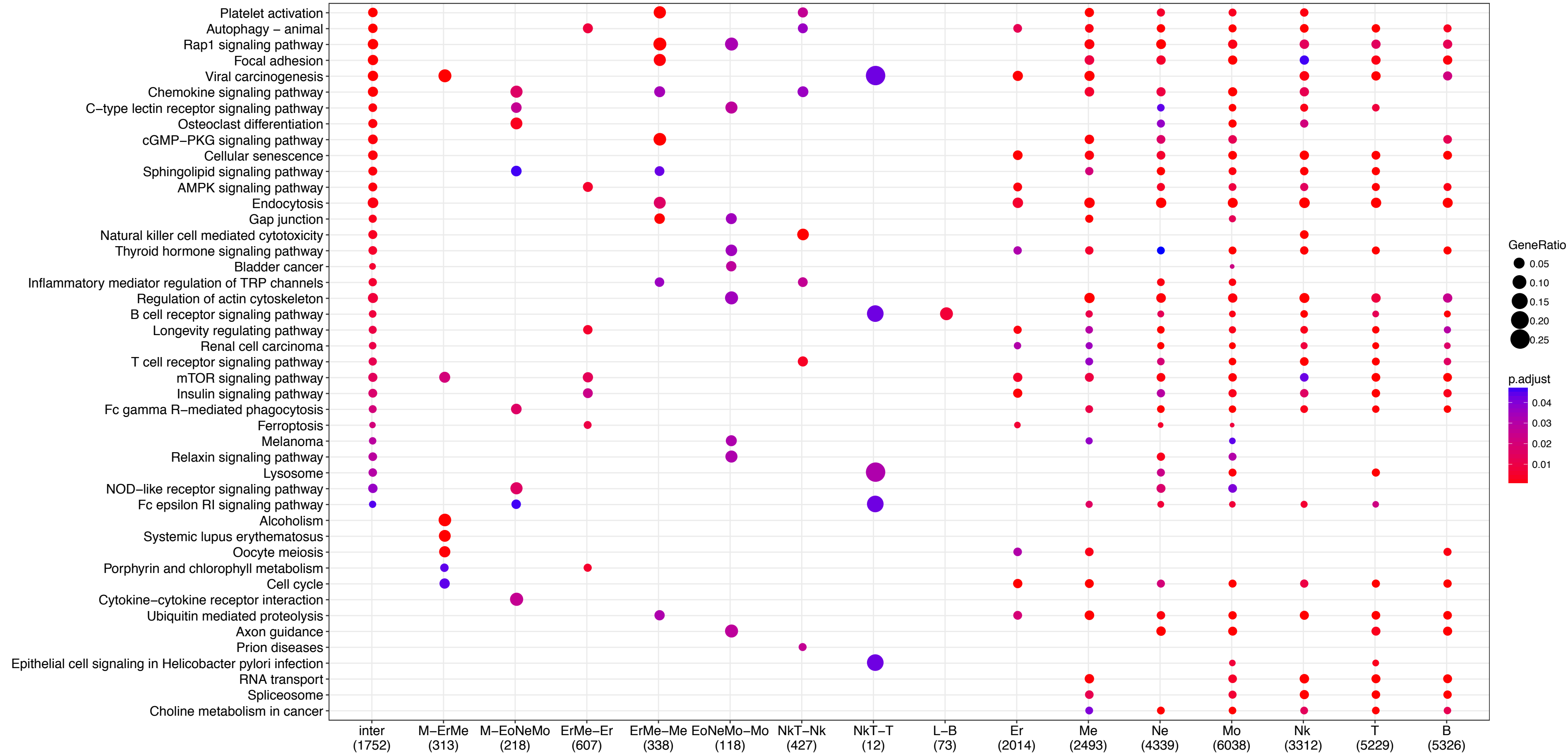

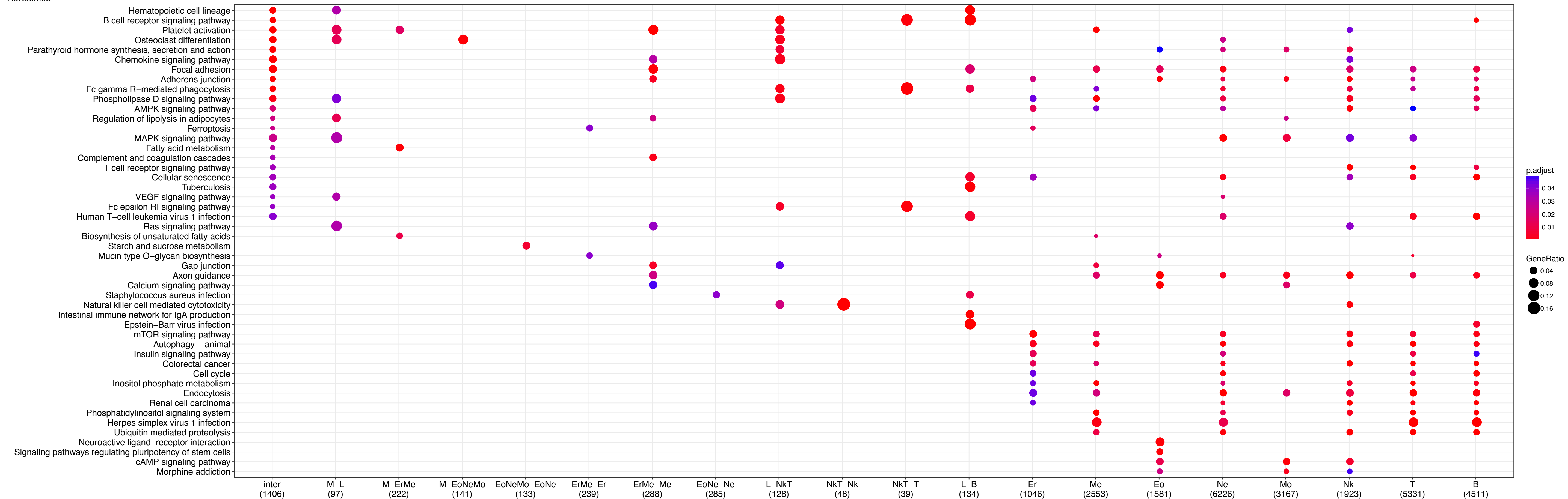

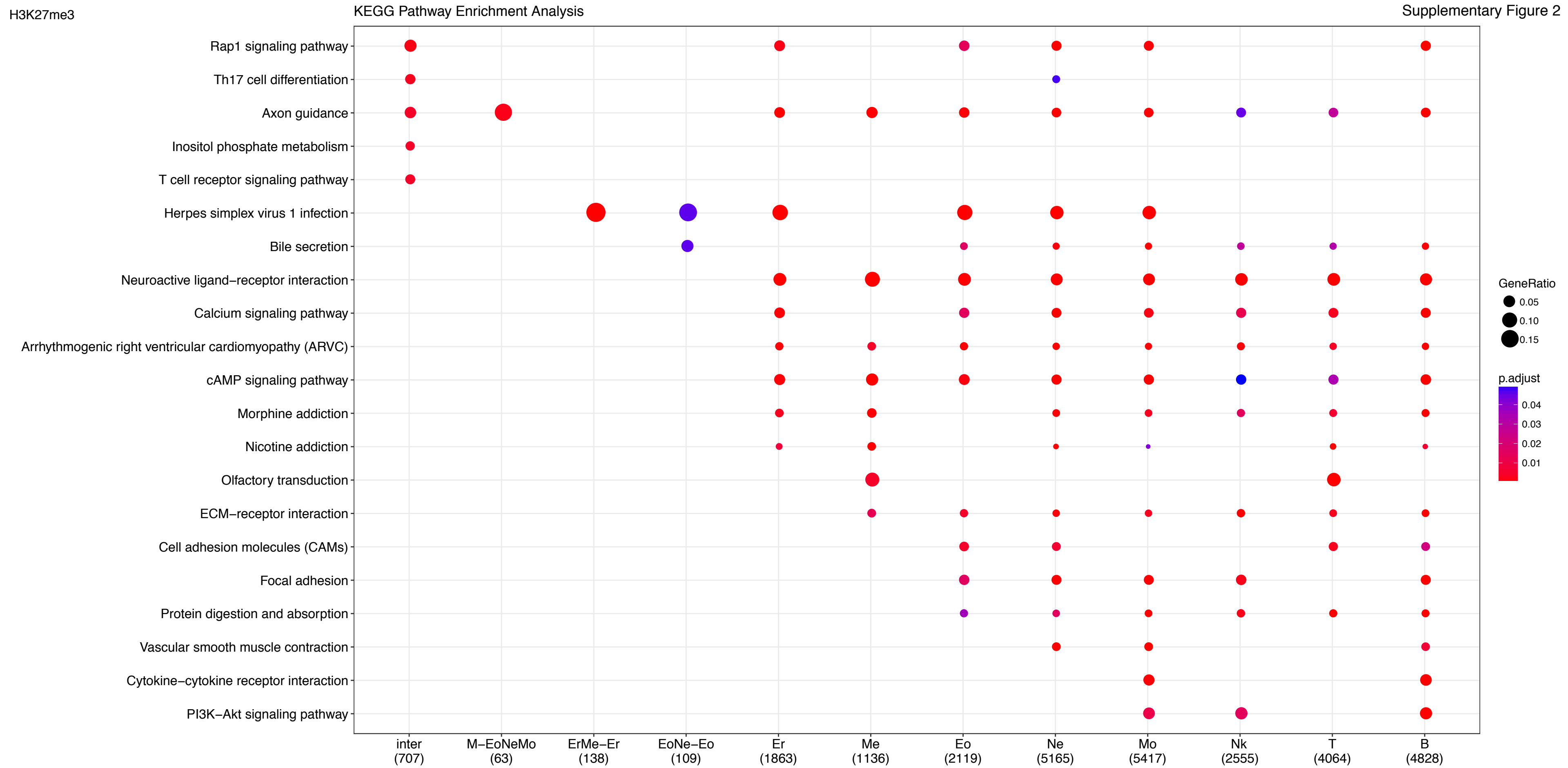

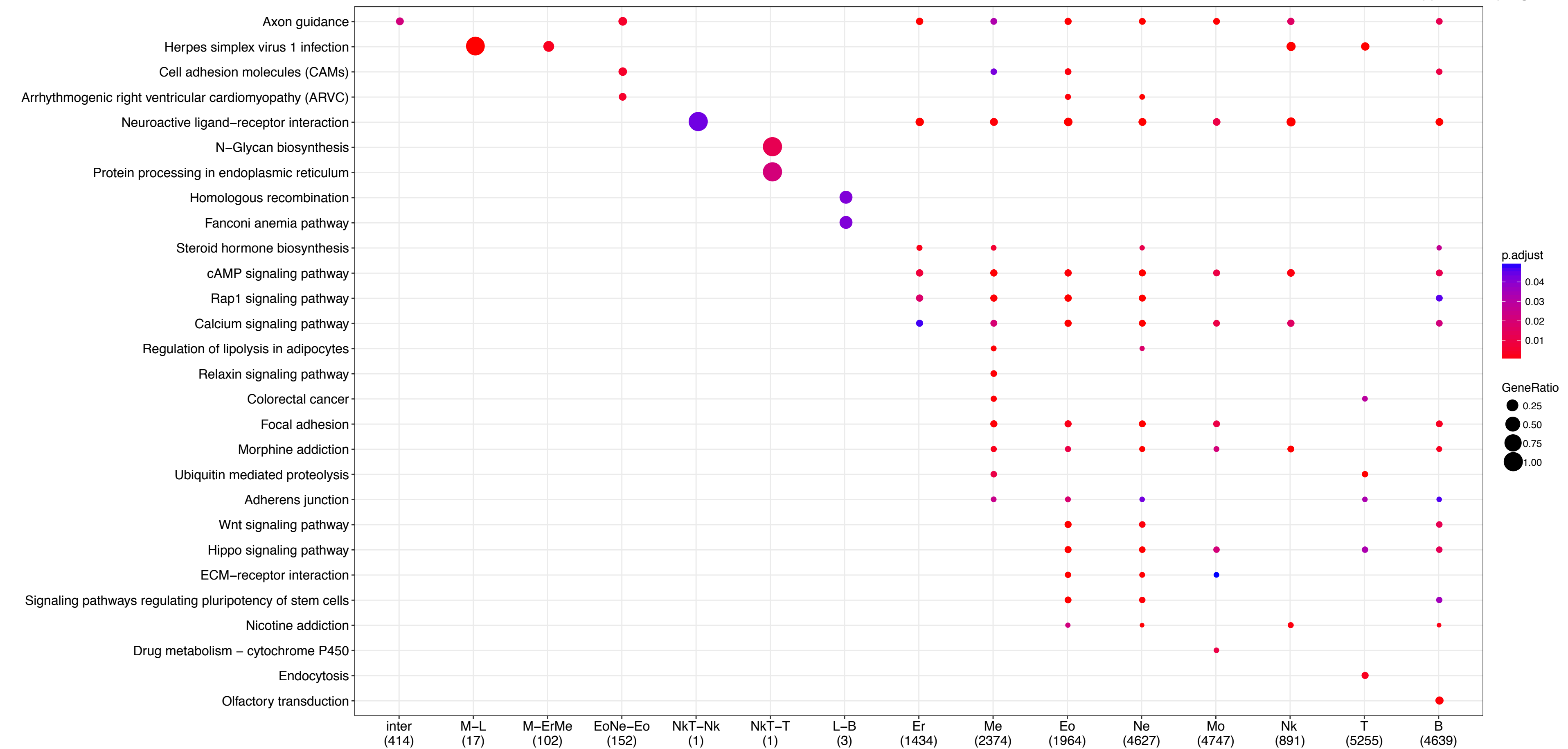

Supplement: Supplementary Fig. 2 — Functional annotation of genes with histone modification changes (ON) for each branch. [file mmc4.pdf]
